# Supplementary material for: Association between age of respiratory syncytial virus infection hospitalization and childhood asthma: A systematic review
Source: PLoS One. 2024 Feb 13;19(2):e0296685. doi: 10.1371/journal.pone.0296685 (PMC10863881; doi:10.1371/journal.pone.0296685)
Supplement: S1 Table — Abbreviations: *: RSV = respiratory syncytial virus. (DOCX) [file pone.0296685.s002.docx]

**S1 Table**. Search terms in each database

| MEDLINE via OVID | Search term |
| --- | --- |
| RSV^*^ infection AND Age AND Child asthma | |
| RSV infection | #1 Respiratory Syncytial Virus Infections/ |
|  | #2 Respiratory Syncytial Viruses/ |
|  | #3 Respiratory Syncytial Virus, Human/ |
|  | #4 Respiratory Syncytial Virus.ab,ti. |
|  | #5 RSV.ab,ti. |
| #1 OR #2 OR #3 OR #4 OR #5 | |
| Age | #6 Age-dependen*.ab,ti. |
|  | #7 Age effect?.ab,ti. |
|  | #8 Age difference*.ab,ti. |
|  | #9 Effect? of age.ab,ti. |
|  | #10 Influence of age.ab,ti. |
|  | #11 (Age adj4 Infection).ab,ti. |
| #6 OR #7 OR #8 OR #9 OR #10 OR #11 | |
| Child asthma | #12 Asthma/ |
|  | #13 Recurrent wheeze.ab,ti. |
| # 12 OR #13 | |
| Embase | Search terms |
| RSV infection AND Age AND Child asthma | |
| RSV infection | #1 'human respiratory syncytial virus'/exp OR 'human respiratory syncytial virus' |
|  | #2 'human respiratory syncytial virus':ab,ti |
|  | #3 'human respiratory syncytial virus'/exp OR 'human respiratory syncytial virus' |
|  | #4 'rsv':ab,ti |
| #1 OR #2 OR #3 OR #4 | |
| Age | #5 'age-dependen*':ab,ti |
|  | #6 'age effect?':ab,ti |
|  | #7 'age difference*':ab,ti |
|  | #8 'effect? of age':ab,ti |
|  | #9 'influence of age':ab,ti |
|  | #10 'influence of age':ab,ti |
| #5 OR #6 OR #7 OR # 8 OR #9 OR #10 | |
| Child asthma | #11 'asthma'/exp |
|  | #12 'recurrent wheez*':ab,ti |
| #11 OR #12 | |
| Central | Search terms |
| RSV infection AND Child asthma | |
| RSV infection | ("respiratory syncytial virus"):ti,ab,kw |
| Child asthma | ("asthma"):ti,ab,kw |
| International Clinical Trials Registry Platform | Search terms |
| RSV infection AND Child asthma | |
| RSV infection | Respiratory Syncytial Viruses |
| Child asthma | Asthma |

Abbreviations: *: RSV = respiratory syncytial virus
