# Supplementary material for: Association between age of respiratory syncytial virus infection hospitalization and childhood asthma: A systematic review
Source: PLoS One. 2024 Feb 13;19(2):e0296685. doi: 10.1371/journal.pone.0296685 (PMC10863881; doi:10.1371/journal.pone.0296685)
Supplement: S2 Table — (DOCX) [file pone.0296685.s003.docx]

**S2 Table**. Signaling questions for quality assessment

| **1. Study Participation** |  |
| --- | --- |
| *Source of target population* | The source population or population of interest is adequately described for key characteristics (LIST). |
| *Method used to identify population* | The sampling frame and recruitment are adequately described, including methods to identify the sample sufficient to limit potential bias (number and type used, e.g., referral patterns in health care) |
| *Recruitment period* | Period of recruitment is adequately described |
| *Place of recruitment* | Place of recruitment (setting and geographic location) are adequately described |
| *Inclusion and exclusion criteria* | Inclusion and exclusion criteria are adequately described (e.g., including explicit diagnostic criteria or  “zero time” description). |
| *Adequate study participation* | There is adequate participation in the study by eligible individuals |
| *Baseline characteristics* | The baseline study sample (i.e., individuals entering the study) is adequately described for key characteristics. |
| **2. Study Attrition** |  |
| *Proportion of baseline sample available for analysis* | Response rate (i.e., proportion of study sample completing the study and providing outcome data) is adequate. |
| *Attempts to collect information on participants who dropped out* | Attempts to collect information on participants who dropped out of the study are described. |
| *Reasons and potential impact of subjects lost to follow-up* | Reasons for loss to follow-up are provided. |
| *Outcome and prognostic factor information on those lost to follow-up* | Participants lost to follow-up are adequately described for key characteristics. |
|  | There are no important differences between key characteristics and outcomes in participants who completed the study and those who did not. |
| **3. Prognostic Factor Measurement** |  |
| *Definition of the PF* | A clear definition or description of 'PF' is provided (e.g., including dose, level, duration of exposure, and clear specification of the method of measurement). |
| *Valid and Reliable Measurement of PF* | Method of PF measurement is adequately valid and reliable to limit misclassification bias (e.g., may include relevant outside sources of information on measurement properties, also characteristics, such as blind measurement and limited reliance on recall). |
|  | Continuous variables are reported or appropriate cut-points (i.e., not data-dependent) are used. |
| *Method and Setting of PF Measurement* | The method and setting of measurement of PF is the same for all study participants. |
| *Proportion of data on PF available for analysis* | Adequate proportion of the study sample has complete data for PF variable. |
| *Method used for missing data* | Appropriate methods of imputation are used for missing 'PF' data. |
| **4. Outcome Measurement** |  |
| *Definition of the Outcome* | A clear definition of outcome is provided, including duration of follow-up and level and extent of the outcome construct. |
| *Valid and Reliable Measurement of Outcome* | The method of outcome measurement used is adequately valid and reliable to limit misclassification bias (e.g., may include relevant outside sources of information on measurement properties, also characteristics, such as blind measurement and confirmation of outcome with valid and reliable test). |
| *Method and Setting of Outcome Measurement* | The method and setting of outcome measurement is the same for all study participants. |
| **5. Study Confounding** |  |
| *Important Confounders Measured* | All important confounders, including treatments (key variables in conceptual model: LIST), are measured. |
| *Definition of the confounding factor* | Clear definitions of the important confounders measured are provided (e.g., including dose, level, and duration of exposures). |
| *Valid and Reliable Measurement of Confounders* | Measurement of all important confounders is adequately valid and reliable (e.g., may include relevant outside sources of information on measurement properties, also characteristics, such as blind measurement and limited reliance on recall). |
| *Method and Setting of Confounding Measurement* | The method and setting of confounding measurement are the same for all study participants. |
| *Method used for missing data* | Appropriate methods are used if imputation is used for missing confounder data. |
| *Appropriate Accounting for Confounding* | Important potential confounders are accounted for in the study design (e.g., matching for key variables, stratification, or initial assembly of comparable groups). |
|  | Important potential confounders are accounted for in the analysis (i.e., appropriate adjustment). |
| **6. Statistical Analysis and Reporting** |  |
| *Presentation of analytical strategy* | There is sufficient presentation of data to assess the adequacy of the analysis. |
| *Model development strategy* | The strategy for model building (i.e., inclusion of variables in the statistical model) is appropriate and is based on a conceptual framework or model. |
|  | The selected statistical model is adequate for the design of the study. |
| *Reporting of results* | There is no selective reporting of results. |
