# Supplementary material for: Association between age of respiratory syncytial virus infection hospitalization and childhood asthma: A systematic review
Source: PLoS One. 2024 Feb 13;19(2):e0296685. doi: 10.1371/journal.pone.0296685 (PMC10863881; doi:10.1371/journal.pone.0296685)
Supplement: S3 Table — *: The number of participants included in the analysis evaluating the association between age at first RSV infection and child asthma inception. (DOCX) [file pone.0296685.s004.docx]

**S3 Table.** Patient characteristics

| **Study (first author and publication date)** | **Male (%)** | **Socioeconomic status** | **Maternal smoking** | **Day-care use** | **Pet exposure** | **Family history of asthma (%)** |
| --- | --- | --- | --- | --- | --- | --- |
| Muñoz-Quiles 2023 | 105,696/214,303 (49) | Not described | Not described | Not described | Not described | Not described |
| Koponen 2012 | 86/166 (52) | Not described | 29/166 (17) | Not described | 51/166 (31) | 34/166 (20) |
| Homaira 2019 | 10,276/18,042 (57) | The index of relative socioeconomic advantage and disadvantage (IRSAD): 1 = 2,301/18,042 (13%); 2 = 2,530/18,042 (14%); 3 = 4,303/18,042 (24%), 4 = 4,550/18,042 (25%); 5 = 4,356/18,042 (24%)  1 as most disadvantaged, 5 as most advantaged | 4,135/18,042 (23) | Not described | Not described | Not described |
| Zhou 2021 | 190/266 (71) | Not described | Not described | Not described | 18 (7) | Not described |
| Wang 2022 | 13,265/23,365 (57) | Scottish index of multiple deprivation (SIMD):  1 = 7,100/23,365 (30%); 2 = 5,232/23,365 (22%); 3 = 4,132/23,365 (18%); 4 = 3,630/23,365 (16%); 5 = 3,271/23,365 (14%)  1 as most deprived, 5 as at least deprived | 7,168/23,365 (31) | Not described | Not described | Not described |

*: The number of participants included in the analysis evaluating the association between age at first RSV infection and child asthma inception
