# Supplementary material for: Association between age of respiratory syncytial virus infection hospitalization and childhood asthma: A systematic review
Source: PLoS One. 2024 Feb 13;19(2):e0296685. doi: 10.1371/journal.pone.0296685 (PMC10863881; doi:10.1371/journal.pone.0296685)
Supplement: S4 Table — (DOCX) [file pone.0296685.s005.docx]

**S4 Table**. QUIPS tool quality assessment of included studies

| **1. Study Participation** | Muñoz-Quiles 2023 | Koponen 2012 | Homaira 2019 | Zhou 2021 | Wang 2022 |
| --- | --- | --- | --- | --- | --- |
| *Source of target population* | Yes | Yes | Yes | Yes | Yes |
| *Method used to identify population* | Yes | No | Yes | No | Yes |
| *Recruitment period* | Yes | Yes | Yes | Yes | Yes |
| *Place of recruitment* | Yes | Yes | Yes | Yes | Yes |
| *Inclusion and exclusion criteria* | Yes | No | Yes | Yes | Yes |
| *Adequate study participation* | Yes | Unclear | Yes | Unclear | Yes |
| *Baseline characteristics* | Yes | Yes | Yes | No | Yes |
| ***Rate of risk of bias*** | **Low** | **Moderate** | **Low** | **Moderate** | **Low** |
| **2. Study Attrition** |  |  |  |  |  |
| *Proportion of baseline sample available for analysis* | No | Yes | No | No | No |
| *Attempts to collect information on participants who dropped out* | No | No | No | No | No |
| *Reasons and potential impact of subjects lost to follow-up* | No | Yes | No | No | No |
| *Outcome and prognostic factor information on those lost to follow-up* | No | No | No | No | No |
|  | No | No | No | No | No |
| ***Rate of risk of bias*** | **High** | **Moderate** | **High** | **High** | **High** |
| **3. Prognostic Factor Measurement** |  |  |  |  |  |
| *Definition of the PF* | Yes | Yes | Yes | Yes | Yes |
| *Valid and Reliable Measurement of PF* | No | Yes | No | Yes | No |
|  | Not applicable | Not applicable | Not applicable | Not applicable | Not applicable |
| *Method and Setting of PF Measurement* | Yes | Yes | Yes | Yes | Yes |
| *Proportion of data on PF available for analysis* | Yes | Yes | Yes | Yes | Yes |
| *Method used for missing data* | No | No | No | No | No |
| ***Rate of risk of bias*** | **Moderate** | **Low** | **Moderate** | **Low** | **Moderate** |
| **4. Outcome Measurement** |  |  |  |  |  |
| *Definition of the Outcome* | Yes | Yes | Yes | No | Yes |
| *Valid and Reliable Measurement of Outcome* | No | Yes | No | No | No |
| *Method and Setting of Outcome Measurement* | Yes | Yes | Yes | No | Yes |
| ***Rate of risk of bias*** | **Moderate** | **Low** | **Moderate** | **High** | **Moderate** |
| **5. Study Confounding** |  |  |  |  |  |
| *Important Confounders Measured* | No | No | No | No | No |
| *Definition of the confounding factor* | Yes | Yes | Yes | Yes | Yes |
| *Valid and Reliable Measurement of Confounders* | Yes | No | Yes | Yes | Yes |
| *Method and Setting of Confounding Measurement* | Yes | No | Yes | Yes | Yes |
| *Method used for missing data* | No | No | No | No | No |
| *Appropriate Accounting for Confounding* | No | No | No | No | No |
|  | No | No | No | Yes | No |
| ***Rate of risk of bias*** | **Moderate** | **High** | **Moderate** | **Moderate** | **Moderate** |
| **6. Statistical Analysis and Reporting** |  |  |  |  |  |
| *Presentation of analytical strategy* | Yes | Yes | Yes | Yes | Yes |
| *Model development strategy* | Yes | Yes | Yes | Yes | Yes |
|  | Yes | No | Yes | No | Yes |
| *Reporting of results* | Yes | Yes | Yes | Yes | Yes |
| ***Rate of risk of bias*** | **Low** | **Moderate** | **Low** | **Moderate** | **Low** |
