# Supplementary material for: Association between age of respiratory syncytial virus infection hospitalization and childhood asthma: A systematic review
Source: PLoS One. 2024 Feb 13;19(2):e0296685. doi: 10.1371/journal.pone.0296685 (PMC10863881; doi:10.1371/journal.pone.0296685)
Supplement: Attachment — Submitted filename: plos_one_response.docx [file pone.0296685.s006.docx]

**Point-by-Point Responses to the Comments of the Reviewers**

**Academic Editor:**

*1. Please ensure that your manuscript meets PLOS ONE's style requirements, including those for file naming. The PLOS ONE style templates can be found at*

*https://journals.plos.org/plosone/s/file?id=wjVg/PLOSOne_formatting_sample_main_body.pdf and*

*https://journals.plos.org/plosone/s/file?id=ba62/PLOSOne_formatting_sample_title_authors_affiliations.pdf.*

**Response:** We have revised the manuscript and confirmed it meets PLOS ONE’s style requirements.

*2. Thank you for stating the following in the Competing Interests section: 
"AS received financial support for his doctoral study from Vanderbilt University Medical Center, Center for Asthma Research and the Fulbright Association, TG is supported by grants from the NIH, TH is supported by grants from the NIH and serves on DSMBs for Pfizer, and as an external scientific consultant for Sanofi."*

*Please confirm that this does not alter your adherence to all PLOS ONE policies on sharing data and materials, by including the following statement: ""This does not alter our adherence to  PLOS ONE policies on sharing data and materials.” (as detailed online in our guide for authors* [*http://journals.plos.org/plosone/s/competing-interests*](http://journals.plos.org/plosone/s/competing-interests)*).  If there are restrictions on sharing of data and/or materials, please state these. Please note that we cannot proceed with consideration of your article until this information has been declared.*

*Please include your updated Competing Interests statement in your cover letter; we will change the online submission form on your behalf.*

**Response:** We have added the statement “T In the Competing Interests section, this does not alter our adherence to PLOS ONE policy on sharing data and materials. In addition, we have updated the competinginterests statement in the cover letter.

*3. Please include captions for your Supporting Information files at the end of your manuscript, and update any in-text citations to match accordingly. Please see our Supporting Information guidelines for more information:* [*http://journals.plos.org/plosone/s/supporting-information*](http://journals.plos.org/plosone/s/supporting-information)*.*.

**Response:** We have revised the manuscript accordingly and confirmed that it adheres to the Supporting Information guidelines.

**Reviewer #1:** *This is a good systemic review which highlights about the relationship between age of RSV infection and asthma risk. Due to the heterogeneous epidemiological designs of the enrolled studies. Although this study could not provide summary statistics nor valid conclusions, it still worth to know by journal readers. Furthermore, a large-scale prospective cohort study or an instrumental variable approach with a valid variable for RSV infection are required to overcome measurement bias and confounding.*

**Response:** We appreciate the reviewer’s recognition of the important question addressed and the gap that this manuscript identifies.

**Reviewer #2:** *It is an interesting study, since it investigates whether there is clear information about the age at which RSV occurs and not hospitalization. I liked the emphasis given to it, it opens up fields of research that have always been suggested, but there are no quality studies.*

**Response:** We are grateful for your thoughtful comments.

**Reviewer #3**: *The current study highlights gaps in our knowledge of whether there is a susceptibility age range during which RSV infection confers the highest risk of later development of asthma and the challenges of designing studies to address this question. The authors assume a large-scale prospective cohort study to address these gaps. However, in my personal opinion, a large-scale prospective cohort as you designed is difficult to implement. Can you design a simple, highly feasible observational study with a relatively simple test method and without high risk of bias to determine the association between RSV infection and asthema?*

**Response:** Thank you for your comment. Although a large-scale prospective cohort study is an ideal study design, we agree with your opinion that it is challenging to implement. Children’s birthday/birth month has been consistently shown to be associated with an increased risk of RSV infection and childhood asthma. We thus propose an instrumental variable approach with date of birth as an instrumental variable to RSV infection. An association between date of birth and asthma/wheeze via and only via RSV infection will provide strong evidence of a causal relationship between RSV infection and asthma. We have added these future study plans in our conclusion section

"To determine the association between age of RSV infection and risk of asthma, a large-scale prospective cohort study will be an ideal study design to overcome measurement bias and confounding. Another option is an instrumental variable approach taking advantage of the well observed association between birth month and asthma. It is unlikely a person’s birthday causes his/her risk for asthma, a formal instrumental analysis demonstrating that the date of birth and asthma association is through and only through increasing the risk of having RSV infection will provide strong evidence of the causal relationship. We are planning to conduct further studies based on these two approaches.” (Page 29, Line 348–351)

Minor aspects:

*1. page 17, lines 236, 237, a repeated sentence “Compared to those without RSV hospitalization”.*

Response: Thank you for pointing it out. We have deleted the repeated phrase.

“The study by Wang et al. estimated the hazard ratios of first asthma hospitalization among those with RSV hospitalizations at age 0–5 months and 6–23, compared to those without RSV hospitalization. ~~Compared to those without RSV hospitalization,~~ C~~c~~hildren with RSV hospitalization at age 6–23 months had a significantly higher hazard ratio of asthma hospitalization during age 4–6 years”

*2. Figure 1, in my opinion, do not draw the flow diagram by yourself, just use the original PRISMA flow diagram by filling your contents.*

**Response:** We have updated the figure using the original PRISMA flow diagram.

Figure 1.
